# Studies on the Tissue-Related Phenotypic Heterogeneity of Murine B Cells

**DOI:** 10.1155/1998/61492

**Published:** 1998

**Authors:** Péter Balogh, Attila Kumánovics, Istvan Juhasz

**Affiliations:** 1 lmmunological and Biotechnological Laboratory University Medical School of Pdcs Hungary; 2 Department of Dermatology University Medical School of Debrecen Hungary

**Keywords:** B cells, spleen, bone marrow, L-selectin, IBL-2, SCID

## Abstract

The development of B cells is accompanied by their ability to specifically enter the peripheral lymphoid tissues. Recently, we described a novel rat monoclonal antibody (IBL-2; IgG2b/*k* reacting with a 26/29-kD heterodimeric structure of the cell surface. This mAb has been found to recognize differentially the peripheral B cells of mice depending on their tissue origin. The majority of splenic B cells as well as the mature B cells in the bone marrow were stained with this mAb, whereas the B lymphocytes isolated from LN or Peyer's patches displayed only negligible reactivity. We extended these observations by analyzing the relationship between the expression of IBL-2 antigen and L-selection on the surface of B-cell precursors in the bone marrow by multiparameter flow cytometry. Within the B220 positive compartment, a significant difference of L-selectin expression could be observed between the various IBL-2-reactive subsets. Furthermore, we investigated whether evidences for the establishment of tissue-associated phenotypic heterogeneity similar to that found in normal mice could be found upon the adoptive transfer of normal unselected splenic lymphocytes into SCID recipients (Spl-SCID). It has been found that a large part of the splenic B cells preserved their IBL-2 reactivity, whereas the LN B cells had lost the IBL-2 antigen in Spl-SCID. These data indicate that the phenotypic difference within the SCID mice may be the result of the migration of B lymphocytes from the spleen toward the lymph nodes, and the altered expression of the IBL-2 antigen correlates with this process.


*Developmental Immunology*, 1998, Vol. 6, pp. 179–185
Reprints available directly from the publisher
Photocopying permitted by license only

© 1998 OPA (Overseas Publishers Association)
N.V. Published by license under the
Harwood Academic Publishers imprint,
part of the Gordon and Breach Publishing Group
Printed in Malaysia

### Studies on the Tissue-Related Phenotypic Heterogeneity of Murine B Cells

PÉTER BALOGH[a*], ATTILA KUMÁNOVICS[a] and ISTVÁN JUHÁSZ[b]

[a]*Immunological and Biotechnological Laboratory, University Medical School of Pécs, Hungary;* [b]*Department of Dermatology, University Medical School of Debrecen, Hungary*

*(Received 6 August 1996; Accepted 12 August 1997; In final form 30 September 1997)*

The development of B cells is accompanied by their ability to specifically enter the peripheral lymphoid tissues. Recently, we described a novel rat monoclonal antibody (IBL-2; IgG2b/$\kappa$) reacting with a 26/29-kD heterodimeric structure of the cell surface. This mAb has been found to recognize differentially the peripheral B cells of mice depending on their tissue origin. The majority of splenic B cells as well as the mature B cells in the bone marrow were stained with this mAb, whereas the B lymphocytes isolated from LN or Peyer's patches displayed only negligible reactivity. We extended these observations by analyzing the relationship between the expression of IBL-2 antigen and L-selection on the surface of B-cell precursors in the bone marrow by multiparameter flow cytometry. Within the B220 positive compartment, a significant difference of L-selectin expression could be observed between the various IBL-2-reactive subsets. Furthermore, we investigated whether evidences for the establishment of tissue-associated phenotypic heterogeneity similar to that found in normal mice could be found upon the adoptive transfer of normal unselected splenic lymphocytes into SCID recipients (Spl-SCID). It has been found that a large part of the splenic B cells preserved their IBL-2 reactivity, whereas the LN B cells had lost the IBL-2 antigen in Spl-SCID. These data indicate that the phenotypic difference within the SCID mice may be the result of the migration of B lymphocytes from the spleen toward the lymph nodes, and the altered expression of the IBL-2 antigen correlates with this process.

*Keywords:* B cells, spleen, bone marrow, L-selectin, IBL-2, SCID

### INTRODUCTION

The spleen is a major target organ for peripheral lymphocyte homing. Its histological architecture is rather complex, partly composed of erythromyeloid regions containing some migrating lymphoid elements. This red pulp is intermingled with lymphoid white pulp that, according to its cellular content, is further subdivided into T- and B-cell compartments (van Ewijk and Nieuwenhuis, 1985). This considerable cellular and functional heterogeneity has significantly hampered the understanding of how the

*Corresponding author.

lymphocytes may localize to specific regions. Whereas the splenic recirculation kinetics of both the T and B cells have been thoroughly studied (Stevens et al., 1982; Pabst and Westermann, 1991; Picker and Butcher, 1992), little is known about the cellular interactions leading to their extravasation and subsequent tissue positioning. The sinus-lining cells and, less likely, the marginal zone macrophages in the spleen have been reported to perform binding functions similar to those of HEV cells (Kraal et al., 1995; Lyons and Parish, 1995). However, the adhesion of of lymphocytes to either of these cells in the spleen appears to be independent of the L-selectin or $\alpha 4 \beta 7$ molecules, the characteristic leukocyte structures for binding to HEV in the lymph nodes and mucosal lymphoid tissues, respectively (Picker and Butcher, 1992; Bradley et al., 1994). It is worthwhile to mention that the splenic but not the bone marrow homing of myeloid precursors has proved to be independent of the interactions between the VCAM-1 and VLA-4 molecules (Papayannopoulou et al., 1995).

The details of the *in situ* migration of lymphocytes subsequent to the extravasation are also largely unknown. It is supposed that various polysaccharides produced by microenvironmental elements may contribute to the lymphoid organization of the spleen (Parish and Snowden, 1985; Kraal et al., 1994). On the other hand, it is not only the microenvironment that influences the lymphocyte migration, but the presence of certain lymphoid elements can also modulate the composition of the mesenchymal scaffolding, according to the results obtained in SCID mice (Yoshida et al., 1993). Under normal circumstances, the splenic entry of lymphocytes can be divided into two subsequent parts. The extravasation into the red pulp is independent from G-protein-coupled lymphocyte membrane components inhibited by pertussis toxin (PTX), whereas the subsequent migration of T and B lymphocytes from the red pulp toward the various compartments of white pulp is likely to be mediated by PTX-sensitive structures (Cyster and Goodnow, 1995). It is also not known whether what kind of phenotypic alterations take place in the lymphocytes upon their splenic lodging or return from the spleen to the circulation.

We have recently reported the isolation and characterization of a novel rat monoclonal antibody IBL-2. This mAb reacts with the mature B cells (identified as lg-positive cells) purified from spleen or bone marrow, whereas the B cells from other peripheral lymphoid tissues were not recognized. The T cells, including the splenic T cells, do not express the antigen (Balogh et al., 1992; Balogh and Kumánovics, 1995). Here we extend these observations by investigating the coexpression of L-selectin and IBL-2 antigens on B-cell precursors in the bone marrow. In addition, we report the result of experiments employing adoptive cell transfer of normal BALB/c splenic lymphocytes into SCID recipients. These experiments were aimed at confirming the differential reactivity of peripheral B cells with IBL-2 mAb determined by their tissue location.

### RESULTS AND DISCUSSION

### Coexpression of IBL-2 Antigen and L-Selectin on B-Cell Precursors in Bone Marrow

Our previous finding was that, employing anti-immunoglobulin antibodies as lineage marker, the reactivity pattern of splenic B cells was quite similar to that of marrow B cells. The majority of these cells of either source also expressed L-selectin (Balogh et al., 1995). In order to determine the simultaneous expression of L-selectin and IBL-2 antigen on immature B-cell precursors, we first depleted the bone marrow cells of cell-bearing Ig on their surface. The residual cells were labeled with an anti-B220 (RA3-6B2), MEL-14, and IBL-2 mAbs. A representative example of these three-color stainings are shown in Figure 1.

The majority of the $B220^{+}$ cells are IBL-2-negative (approximately 58%), whereas the rest are either weakly or strongly reactive with this mAb (approximately 32% and 10%, respectively). This is in contrast with our previous finding that the Ig-positive bone marrow cell subset contained considerably higher frequency of IBL-$2^{lo}$ cells, ranging between the 60-70% of total (mature) B cells (Balogh et al., 1995) The B220$^-$ population could be resolved into two strikingly different subsets: one is IBL-2-negative, whereas the other one was found to react with this antibody very intensely (IBL-2$^{hi}$). Slightly more than 50% of the latter cells also expressed the L-selectin (bottom right panel) at a detectable level. As the B-cell precursors express the B220 antigen rather early in their development, however, it is likely that the majority of B220$^-$ population is composed of non-B lineage cells.

Although the expression patterns of L-selectin by B220$^+$/IBL-2$^-$ and B220$^+$/IBL-2$^{lo}$ populations do not differ from each other significantly (top middle and right panels), there is an almost threefold increase of the MEL-14 positive subset within the B220$^+$/IBL-2$^{hi}$ compartment (bottom middle panel). The question may arise whether the L-selectin molecule is functional on these B220$^+$/IBL-2$^{lo}$ or B220$^+$/IBL-2$^{hi}$ cells. It has been demonstrated that even though the granulocytic cells may become MEL-14 positive upon the *in vitro* culture of their L-selectin negative precursors induced by a variety of soluble factors, they are unable to bind to HEV cells, presumably due to the lack of other adhesion molecules (Salmi and Jalkanen, 1992).

To our knowledge, there are no data concerning the L-selectin expression during the various stages of murine B-cell differentiation in the bone marrow. Therefore, we can only extrapolate from data obtained in humans, indicating that the expression of CD62L is restricted to the the later phase of medullary B-cell differentiation (Kansas and Dailey, 1989).

### IBL-2 Phenotype of Peripheral B Cells in SCID Transplanted with Normal Spleen Cells

Our previous finding indicated the location-dependent reactivity of the IBL-2 mAb on B cells from various peripheral lymphoid organs of normal lymphopoietic

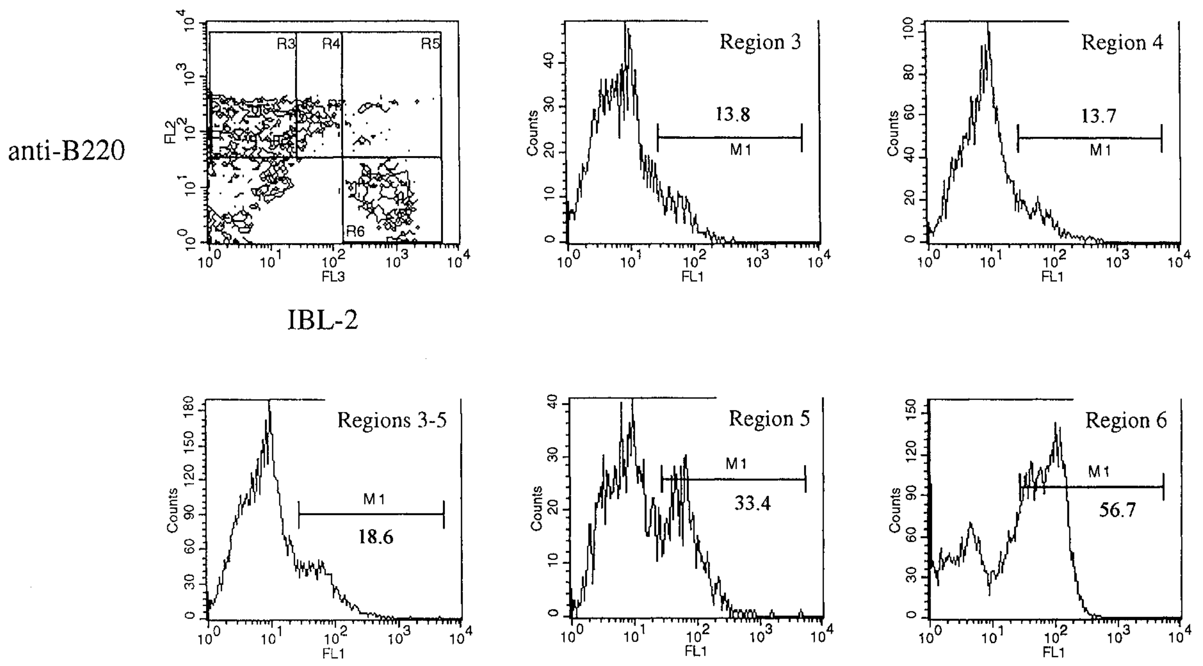

FIGURE 1 Relationship between the expression of L-selectin and IBL-2 antigen on B-cell precursors in the bone marrow. Adult bone marrow cells depleted of IgM-positive cells were stained with anti-B220 and IBL-2 and MEL-14 mAbs labeled with different fluorochromes as described in Materials and Methods. The various regions (3-6) shown on the contour plot (gated on lymphocytes) were used to analyze the expression of L-selectin by the different subsets (histograms). The numbers at the regions indicate the percentage of positive cells obtained by an irrelevant control rat IgG.

mice. In our subsequent studies, we used SCID mice as recipients to study the possible phenotypic alterations affecting the IBL-2 antigen expression of B cells upon their migration from the spleen to the lymph nodes. BALB/c-derived splenic cells as IBL-2-positive cell source was employed. Three days after the intravenous administration of lymphocytes, different lymphoid organs of the recipients were assayed for the IBL-2 profile of B cells (identified as IgM-positive cells). We found this time point as the earliest to allow lymphocytes to be obtained from lymph nodes (LN) in sufficent amount for flow cytometry.

By the end of this period, the overwhelming majority of B cells recovered from the recipients LNs had become IBL-2-negative, even though the original spleen-cell suspension contained a significant amount of IBL-$2^{lo}$ B cells. On the other hand, a significant proportion of B cells of the hosts spleens still retained the IBL-2 antigen on their surface. It is noteworthy that, compared to BALB/c control spleens, the IBL-2-negative B-cell subset slightly increased, and the size of the IBL-$2^{hi}$ compartment was also reduced (Table I). The reasons for these discrepancies are unclear. Our hypothesis is that it probably reflects the difference in the lymphopoietic homeostasis of the spleen between the normal mice and Spl-SCID. In normal mice, the splenic influx of B cells is maintained from both the *de novo* formed B cells released from the bone marrow that are relatively immature (Allman et al., 1993) and fully mature B cells returning from other peripheral lymphoid organs. In Spl-SCID mice, the former source is negligible, whereas a considerable portion of B cells inoculated may have recirculated several times during the 3-day period, taking into account the relatively fast speed of splenic homing (Brelinska et al., 1984; Willfuhr et al., 1989). The T cell remained IBL-2-negative.

The dual staining of splenocytes with an anti-CD45 and IBL-2 mAbs revealed three main populations: $CD45^+$/IBL-$2^-$, $CD45^+$/IBL-$2^{lo}$, and $CD45^{lo}$/IBL-$2^{hi}$. A small, but consistently detectable subset expressed the $CD45^+$/IBL-$2^{hi}$ phenotype (not shown). Of these, the $CD45^{lo}$/IBL-$2^{hi}$ subset may represent some extramedullary erythroid-committed progenitors (manuscript in preparation). The origin of IBL-$22^{hi}$ non-B cells (donor/recipient) in the transplanted animals was not further investigated.

The control SCID mice did not contain detectable number of B cells, even though some Thy-1-positive cells could be observed. Another finding was that they also almost completely lack the IBL-$2^{lo}$ population, whereas the IBL-$2^{hi}$ subset is clearly present, approximately at the same frequency as in normal BALB/c mice (not shown).

The tissue distribution of the IBL-2-reactive elements in the spleen appeared to be similar in both the transplanted and control SCID mice (Figure 2). The strongly positive cells tend to form a ring around the white pulp, where the cells are arranged in tight clusters containing around 20 to 70 cells. The similarity of immunohistochemical staining of the spleen of the control and injected SCID mice with IBL-2 mAb together with FACS data indicate that the majority of these bright cells are of non-B lineage. However, at the interpretation of the negative immunohistochemical staining of a B-cell area, one must take into account that, according to our quantitative FACS data, the level of IBL-2 antigen expression on

TABLE I Distribution of IBL-2-Reactive B Cells in SCID Mice

| Cell sample analyzed | IBL-2-negative | IBL-$2^{lo}$ | IBL-$2^{hi}$ |
|---|---|---|---|
| BALB/c spleen | 34.7 ± 4.2 | 47.1 ± 3.2 | 17.9 ± 3.7 |
| BALB/c lymph node | 94.4 ± 3.4 | 2.7 ± 0.7 | 2.1 ± 0.6 |
| Spl-SCID spleen | 57.3 ± 4.2 | 38.3 ± 4.7 | 4.7 ± 1.3 |
| Spl-SCID lymph node | 91.7 ± 6.4 | 3.4 ± 1.4 | 1.9 ± 0.8 |

*Note*: The results of spleen are the average ± SD of five mice analyzed individually; the lymph node results are the average ± SD of pooled lymph node suspensions from four mice. The B cells were identified using biotinylated anti-IgM antibodies.

### Monoclonal Antibodies supernatant. After 1-hr incubation at room temperature, the specimens were washed and further incubated in the dark with FITC-conjugated affinity-purified sheep anti-rat IgG antibodies. After washing, the specimens were mounted with PBS-glycerol and viewed under a Nikon FXA epifluorescent microscope.

### Depletion of B Cells from the Bone Marrow

The bone marrow cells were depleted of mature B cells by using rosette technique. Briefly, the bone marrow cells were incubated with rat anti-mouse IgM mAb (Serotec, UK) followed by washing. The cells were further incubated with sheep erythrocytes (SRBC) precoated with affinity-purified sheep anti-rat IgG. The mixture was incubated at 4°C, with constant rotation in PBS contatinig 0.1% BSA and Na-azide. The rosetted cells were rerosetted by adding SRBC precoated with rat IgG (Hunt, 1986).

### Flow Cytometric Analyses

The flow cytometric analyses were performed on cell suspensions from various lymphocyte sources using a cocktail of monoclonal antibodies. For two-color staining, the cells were incubated with IBL-2 supernatant, followed by FITC-conjugated anti-rat IgG. The residual binding sites of the secondary antibodies were blocked by normal rat serum. After washing, biotinylated rat mAbs (anti-mouse Thy-1, anti-mouse IgM [LO-MM-9, Serotec] anti-mouse CD45) were added, that were subsequently detected with PE-streptavidin (Sigma).

The expression of IBL-2 antigen correlated with that of L-selectin on B cells and on their precursors in the bone marrow was detected using a three-color staining procedure. Normal bone marrow or spleen cells were incubated with PE-labeled anti-mouse CD45RA mAb as a lineage marker and biotinylated IBL-2 mAb, which was revealed using CyChrome-streptavidin (PharMingen, USA). The appearance of L-selectin on the B220-positive compartment was determined by their subsequent staining with FITC-labeled MEL-14 mAb. After staining, the cells were fixed in 1% buffered paraformaldehyde. The samples were run on a Becton-Dickinson FACSCalibur flow cytometer and analyzed with the CellQuest software package.

### *Acknowledgements*

The authors wish to thank Dr. Katalin Pálóczi (National Institute for Haematology and Immunology, Budapest) for her generosity in providing access to a flow cytometer. This work was supported by OTKA Grants No. F 016695 (P.B.) and T 16978 (I.J.)

### *References*

Allman D.M., Ferguson S.E., Lentz V.M., and Cancro M.P. (1993). Peripheral B cell maturation II. Heat-stable antigen$^{hi}$ splenic B cells are an immune developmental intermediate in the production of long-lived marrow-derived B cell. J. Immunol. **151**: 4431-4444.

Balogh P., Bebők Zs, and Németh P. (1992). Cellular enzyme-linked immunocircle assay. A rapid assay of hybridomas produced against cell surface antigens. J. Immunol. Meth. **153**: 141-150.

Balogh P., Szekeres Gy, and Németh P. (1994). Hapten-mediated identification of cell membrane antigens using an anti-FITC monoclonal antibody. J. Immunol. Meth. **169**: 35-40.

Balogh P., and Kumánovics A. (1995). Tissue-associated phenotypic heterogeneity of peripheral B cells in mice. Immunology **86**: 560-567.

Bradley L.M., Watson S.R., and Swain S.L. (1994). Entry of naive CD4 T cells into peripheral lymph nodes requires L-selectin. J. Exp. Med. **180**: 2401-2406.

Brelinska R., Pilgrim C., and Reisert I. (1984). Pathways of lymphocyte migration within the periarteriolar lymphoid sheath of rat spleen. Cell. Tissue Res. **236**: 661-667.

Cyster J.G., and Goodnow C.C. (1995). Pertussis toxin inhibits migration of B and T lymphocytes into splenic white pulp cords. J. Exp. Med. **182**: 581-586.

Hunt S.V. (1986). Preparative immunoselection of lymphocyte populations. In Handbook of Experimental Immunology, Weir D.M., et al., Eds. Oxford: Blackwell), pp. 55.8-55.12.

Kansas G.S., and Dailey M.O. (1989). Expression of adhesion structures during B cell development in man. J. Immunol. **142**: 3058-3072.

Kraal G., Hoeben K., Breve J., and van den Berg T.K. (1994). The role of sialic acid in the localization of lymphocytes in the spleen. Immunobiology **190**: 138-149.

Kraal G., Schornagel K., Streeter P.R., Holzmann B., and Butcher E.C. (1995). Expression of the mucosal vascular addressin, MAdCAM-1, on sinus-lining cells in the spleen. Am. J. Pathol. **147**: 763-771.

Lyons A.B., and Parish C.R. (1995). Are murine marginal-zone macrophages the splenic white pulp analog of high endothelial venules? Eur. J. Immunol. **25**: 3165-3172.

Mason D.W., Penhale W.J., and Sedgwick J.D. (1987). Preparation of lymphocyte subpopulations. In Lymphocytes: A practical approach, Klaus G. G. B. Ed., (Oxford: IRL Press), p. 35.

Pabst R., and Westermann J. (1991). The role of the spleen in lymphocyte migration. Scanning Microsc. **5**: 1075-1079.

Papayannopoulou T., Craddock C., Nakamoto B., Priestley G.V., and Wolf N.S. (1995). The VLA4/VCAM-1 adhesion pathway defines contrasting mechanisms of lodgement of transplanted murine hemopoietic progenitors between bone marrow and spleen. Proc. Natl. Acad. Sci. USA **92**: 9647-9651.

Parish C.R., and Snowden J.M. (1985). Lymphocytes express a diverse array of specific receptors for sulfated polysaccharides. Cell. Immunol. **91**: 201-214.

Picker L.J., and Butcher E.C. (1992). Physiological and molecular mechanisms of lymphocyte homing. Annu. Rev. Immunol. **10**: 561-591.

Salmi M., and Jalkanen S. (1992). Regulation of L-selectin expression on cultured bone marrow leukocytes and their precursors. Eur. J. Immunol. **22**: 835-843.

Stevens S.K., Weissman I.L., and Butcher E.C. (1982). Differences in the migration of B and T lymphocytes: Organ-selective localization in vivo and the role of lymphocyte-endothelial cell recognition. J. Immunol. **128**: 844-851.

Van Ewijk W., and Nieuwenhuis P. (1985). Compartments, domains and migration pathways of lymphoid cells in the splenic pulp. Experentia **41**: 199-200.

Weston S.A., and Parish C.R. (1992). Calcein: A novel marker for lymphocytes which enter lymph nodes. Cytometry **13**: 739-749.

Willfuhr K.U., Westermann J., and Pabst R. (1989). Absolute numbers of lymphocyte subsets migrating through the compartments of the normal and transplanted rat spleen. Eur. J. Immunol. **20**: 903-911.

Yoshida K., Matsuura N., Tamahashi N., and Takahashi T. (1993). Development of antigenic heterogeneity in the splenic meshwork of severe combined immunodeficient (SCID) mice after reconstitution with T and B lymphocytes. Cell. Tissue. Res. **272**: 1-10.

